# Variables associated with clinical outcomes and switching from unilateral to bitemporal electroconvulsive therapy: a retrospective study

**DOI:** 10.3389/fpsyt.2025.1758353

**Published:** 2026-01-22

**Authors:** Erika Sordo, Louise Fuet, Federica Porpiglia, Marwa Zrelli, Mickaël Amagat, Pierre De Maricourt, Raphaël Gaillard, Sarah Smadja, Fabien Vinckier, Caroline Schimpf, Françoise Tomberli, Aurélien Mazeraud, Philippe Domenech, Moussa A. Chalah

**Affiliations:** 1 Institut de Neuromodulation, Service Hospitalo-Universitaire, Pôle Hospitalo-Universitaire Psychiatrie Paris 15, Hôpital Sainte-Anne, GHU Paris Psychiatrie et Neurosciences, Paris, France; 2 Secteur 15-16, Pôle Hospitalo-Universitaire Psychiatrie Paris 15, GHU Paris Psychiatrie et Neurosciences, Hôpital Sainte-Anne, Paris, France; 3 Service Hospitalo-Universitaire, Pôle Hospitalo-Universitaire Psychiatrie Paris 15, Hôpital Sainte Anne, GHU Paris Psychiatrie et Neurosciences, Paris, France; 4 Université Paris Cité, Paris, France; 5 Service d’Anesthésie Réanimation, Pole Neuro, GHU Paris Psychiatrie et Neurosciences, Paris, France; 6 Computational Brain Team, UNICOG, Neurospin, INSERM-CEA, Paris, France; 7 Gilbert and Rose-Marie Chagoury School of Medicine, Lebanese American University, Byblos, Lebanon

**Keywords:** bitemporal, electroconvulsive therapy, seizure threshold, titration, ultrabrief pulse, unilateral

## Abstract

**Background:**

Electroconvulsive therapy (ECT) remains the most effective treatment for many patients with severe and/or resistant psychiatric disorders. Right unilateral (RUL) ECT, particularly when administered with titration and ultrabrief pulses, provides cognitive advantages compared with bitemporal (BT) ECT without compromising efficacy. However, some patients fail to improve and require switching to BT ECT. The present study aims to evaluate variables associated with efficacy and tolerability during RUL ECT and, when needed, after switching to BT ECT, aiming to identify factors linked to better outcomes with each placement.

**Methods:**

A retrospective review was conducted on 58 adult inpatients treated with RUL ECT. Patients without improvement after 4–6 sessions could be switched to BT ECT. Demographic, clinical, pharmacological, and electric seizure-related data were collected. Treatment response was classified as total, partial, or none. Tolerability was assessed based on common side effects. Group comparisons were performed between RUL and BT ECT periods, and between unswitched and switched patients. Supplementary analysis was conducted to assess the relationship between efficacy/tolerability and the studied variables.

**Results:**

Of the patients who began with RUL ECT, 18 (31%) were switched to BT ECT. Remission occurred in 40% with RUL ECT and reached 55% cumulatively after BT ECT. Adverse effect rates were comparable between groups. Compared to patients who continued with the RUL ECT, those requiring switching had more prior manic episodes (p < 0.05), higher current antipsychotic use (p < 0.05), and a tendency for ECT to be indicated more often for severity than for treatment resistance (p < 0.10). Within the switched subgroup, clozapine use and ECT charge increased during BT sessions compared to the RUL course (p < 0.05).

**Conclusions:**

Initiating treatment with RUL ECT and transitioning to BT ECT when necessary offers a pragmatic balance between tolerability and efficacy. Certain clinical variables may guide clinicians in anticipating the need for switching from a RUL to a BT setup.

## 1 Introduction

Electroconvulsive therapy (ECT) remains the most effective treatment for severe or resistant psychiatric disorders such as major depressive disorder (MDD), bipolar disorder (BD), psychotic disorders, and catatonia (1, 2). Safety improved early in the history of ECT with the introduction of anesthesia and muscle relaxation (3), yet the optimal protocol remains to be clarified. Bilateral (bitemporal [BT] or bifrontal) ECT is widely used and considered highly effective, producing rapid effects but carrying a higher risk of cognitive adverse effects, particularly memory disturbance (1, 2, 4). Right unilateral (RUL) ECT offers a more favorable cognitive profile and comparable efficacy when delivered at high doses, although some studies reported slower or less consistent improvement than BT (1, 5–15). Other determinants include the method for determining the electric charge (age-based method [ABM] *vs*. titration method [TM]) and the pulse width (ultrabrief [UBP] *vs*. brief [BP]) (1, 16), with best cognitive tolerability achieved combining TM, RUL, and UBP (2, 4, 7, 17–21).

Despite its efficacy, ECT remains underused due to stigma and cognitive risks, reinforcing the need for best practices to optimize acceptability and outcomes (2, 22). International guidelines (e.g., Canadian Network for Mood and Anxiety Treatments [CANMAT]; 1) recommend starting with RUL and switching to BT if response is insufficient. A proportion of patients ultimately require such a switch to achieve remission (11, 14, 23–25). This sequential approach balances efficacy and tolerability, though predictors of RUL non-response and optimal switching remain unclear.

In France, BT with ABM has been the usual practice (26). At Sainte Anne Hospital - the largest psychiatric hospital in Paris performing more than 2700 ECT sessions yearly (3168 in 2024) - practices shifted in September 2023 when the Institute of Neuromodulation (INM) introduced TM, RUL, and UBP (0.3 ms) instead of ABM, BT, and near-UBP (0.5 ms), in line with international recommendations (1). If no signs of clinical improvement are seen after 4–6 sessions compared to the patient’s baseline, retitration could be performed in order to continue the ECT course with BT and BP (1 ms). ABM and BT were reserved for urgent cases. Systematic charge escalation was discouraged across the sessions, except after three consecutive poor-quality seizures following a good one according to Nobler’s criteria (27). In this case a 10–20% increase is allowed. A previous INM study showed that this protocol (TM, RUL, UBP) reduced the frequency of memory disturbance by 23%, while maintaining efficacy compared with earlier practices (2).

Few studies have examined predictors of ECT outcomes (28–31), and even fewer investigated sequential response to different ECT parameters (4, 11, 32). In particular, little is known about factors predicting inadequate RUL response requiring a switch to BT ECT. The present study therefore evaluated variables associated with efficacy and tolerability during RUL ECT and, when needed, after switching to BT ECT, aiming to identify factors linked to better outcomes with each placement.

The primary objective was to assess the relationship between patient-related and ECT-related variables (clinical and sociodemographic factors, administered charge, seizure duration, PIS) and clinical outcomes (efficacy and tolerability) during RUL ECT, and after a switch to BT in non-responders. Secondary objectives were (1) to compare outcomes between RUL and BT courses in patients who underwent the RUL-BT switch, and (2) to compare variables according to treatment outcomes (efficacy and tolerability) within each course. The study thus aimed to help identify patient profiles more likely to benefit from switching and refine evidence-based strategies for optimizing electrode placement and treatment sequencing.

## 2 Materials and methods

This retrospective, monocentric observational study was conducted by the INM at Sainte-Anne Hospital, *GHU Paris Psychiatrie & Neurosciences*, Paris, France. It included patients treated with ECT under the new protocol (RUL, UBP, TM) between September 1, 2023, and June 30, 2025. Treatment procedures followed international ECT guidelines. Data collected during routine care were retrospectively reviewed and anonymized to ensure confidentiality. The study was conducted in accordance with French regulations on retrospective studies, and the Declaration of Helsinki. Informed consent was obtained from patients and/or their legal representatives before treatment initiation.

### 2.1 Participants

The sample included 58 hospitalized patients who underwent an acute ECT course. Inclusion criteria were age ≥ 18 years and admission to the psychiatric units of the *Pôle Hospitalo-Universitaire Paris 15* to receive acute ECT for a psychiatric indication using TM and RUL electrode placement. Exclusion criteria were: urgent clinical presentation requiring acute BT ECT with ABM (as determined by the referring psychiatrist), contraindication to ECT or general anesthesia (as determined by the anesthesiologist), or receipt of consolidation/maintenance rather than acute ECT.

### 2.2 Treatment protocol

ECT sessions were performed with the MECTA Spectrum 5000M or MECTA ∑igma (MECTA Corporation, Portland, OR, USA), delivering pulsed alternating currents (11–1152 mC; 800 mA; 20–120 Hz) with pulse widths of 0.3 or 1 ms. Anesthesia consisted of etomidate (0.15 mg/kg) or, rarely, propofol. Neuromuscular blockade was performed using suxamethonium (0.5 mg/kg). Assisted hyperventilation was performed using a balloon mask. In the first RUL course (UBP: 0.3 ms, d’Elia placement, suprathreshold dose: 6 × seizure threshold [ST]), ST was defined as the lowest dose inducing a seizure ≥20 s, determined by TM titration tables (2, 5, 8, 27, 33). Dose adjustment followed previously described procedures aimed at avoiding unnecessary escalation (2). If no signs of clinical improvement compared to the patient’s baseline – based on clinician’s judgment and reported in the medical charts – were seen after 4–6 sessions, switching to a BT course was possible after a new titration (BP: 1 ms, 1.5 × ST) using TM (2, 5, 8, 27, 33).

Treatment frequency was 2 sessions/week for treatment-resistant cases and up to 3/week for some severe cases, with the number of sessions individualized. Concomitant pharmacological adjustments followed standard recommendations (2, 34–38): antidepressants and antipsychotics were continued; clozapine was given the night before but not the morning of ECT; lithium was withheld the night before and the morning of ECT; benzodiazepines were tapered or substituted with short half-life agents and omitted the evening and morning before ECT; anticonvulsants were discontinued unless needed for epilepsy, with lamotrigine considered compatible. Patients abstained from tobacco and alcohol for at least 12 h before sessions.

Sessions were monitored with scalp electroencephalography (EEG). The INM organized regular training for physicians performing ECT sessions and interpreting EEG to ensure that readings are standardized according to Nobler’s criteria. Additionally, a physician from the INM was present during ECT sessions to supervise the readings.

According to Nobler’s criteria, a good-quality seizure induced by ECT lasts at least 20 s and consists of a generalized seizure with a characteristic electroencephalographic architecture occurring as follows: (a) an epileptic recruiting rhythm phase; (b) high-frequency spike and polyspike activity, referred to as the polyspike phase; (c) a transition to high-amplitude spike-and–slow-wave activity, known as the slow-wave phase; and (d) a subsequent decrease in the amplitude and/or frequency of the slow waves, culminating in a complete and abrupt postictal suppression (PIS) of bioelectric activity (27, 39–41). With respect to seizure termination, the endpoint may be difficult to determine or may be characterized by suppression ranging from poor, to good but progressive, to the optimal pattern of abrupt PIS (41–43).

Patients were evaluated frequently prior to, around the fourth to sixth sessions, and at the end of the ECT course (RUL *vs*. BT). Side effects were monitored clinically and by patient report.

Clinical and sociodemographic data (age, sex, diagnosis, illness duration since onset or diagnosis, ECT indication [severity *vs*. resistance], number of prior hypomanic/manic/depression/psychotic episodes, psychiatric and addictive comorbidities, treatments, current episode duration) and stimulation/seizure outcomes (number of sessions per course, charge, seizure duration, PIS) were extracted from records. Diagnoses were based on DSM-5-TR criteria (44). Δ charge was calculated as the charge difference between first and last session within each ECT course.

Clinical evaluations were conducted by the patients’ referring physicians during hospitalization and assessed both efficacy and tolerability. A categorical assessment of efficacy was performed based on clinicians’ judgment and reflected the response to treatment relative to each patient’s pre-ECT baseline (45). Consistent with prior studies, responses were defined as follows: (a) total response or remission, indicating complete symptom resolution; (b) partial response, reflecting noticeable but insufficient or incomplete clinical improvement with persistent symptoms (i.e., greater than non-response but less than total response); and (c) non-response, defined as no observable clinical improvement (46; 47; 48). Tolerability consisted of assessing the occurrence of side effects (memory disturbance, bradycardia, confusion, and headache). For the purposes of this study, in addition to analyzing each side effect, global tolerability was additionally classified as good when no side effects were reported and poor when one or more side effects occurred during treatment. All these data were documented by physicians in the patients’ medical records.

### 2.3 Statistical analysis

Analyses were performed with IBM SPSS Statistics 30 (IBM Corp., Armonk, NY, USA) in case of group comparisons and GraphPad Prism 10 (version 10.6.1, Boston, Massachusetts USA) in case of logistic regressions. Continuous variables were expressed as mean ± SD and categorical variables as frequencies and percentages. As data were not normally distributed, non-parametric tests were used. *χ*² was applied for categorical comparisons (Fisher exact test applied for confirmation when appropriate). First, between-group comparisons were performed between patients who completed only the first ECT course (n=40) and those who switched to a second course (n=18) using the Mann-Whitney test. Second, within-group comparisons between the first and second courses in patients who underwent the switch (n=18) were conducted with the Wilcoxon signed-ranked test. Third, in each ECT course, exploratory supplementary analyses were performed: the study outcomes were compared according to clinical response (total *vs*. partial *vs*. non-response; Kruskal-Wallis test) and tolerability (good *vs*. poor; Mann-Whitney test). Effect sizes were estimated for the Mann–Whitney and the Wilcoxon signed-rank test using r =|*Z|*/√*n* and for the Kruskal–Wallis test using E^2R^=*H*/[(*n*^2^ − 1)/(*n* + 1)], where *Z* and *H* are the respective test statistics and *n* is the number of observations (49). Given the small sample size, no statistical corrections for multiple comparisons were performed. Finally, logistic regression analyses were performed to identify predictors of switching among variables that differed significantly in group comparisons. Statistical significance was set at p < 0.05.

## 3 Results

### 3.1 Descriptive data

**Figure 1:**
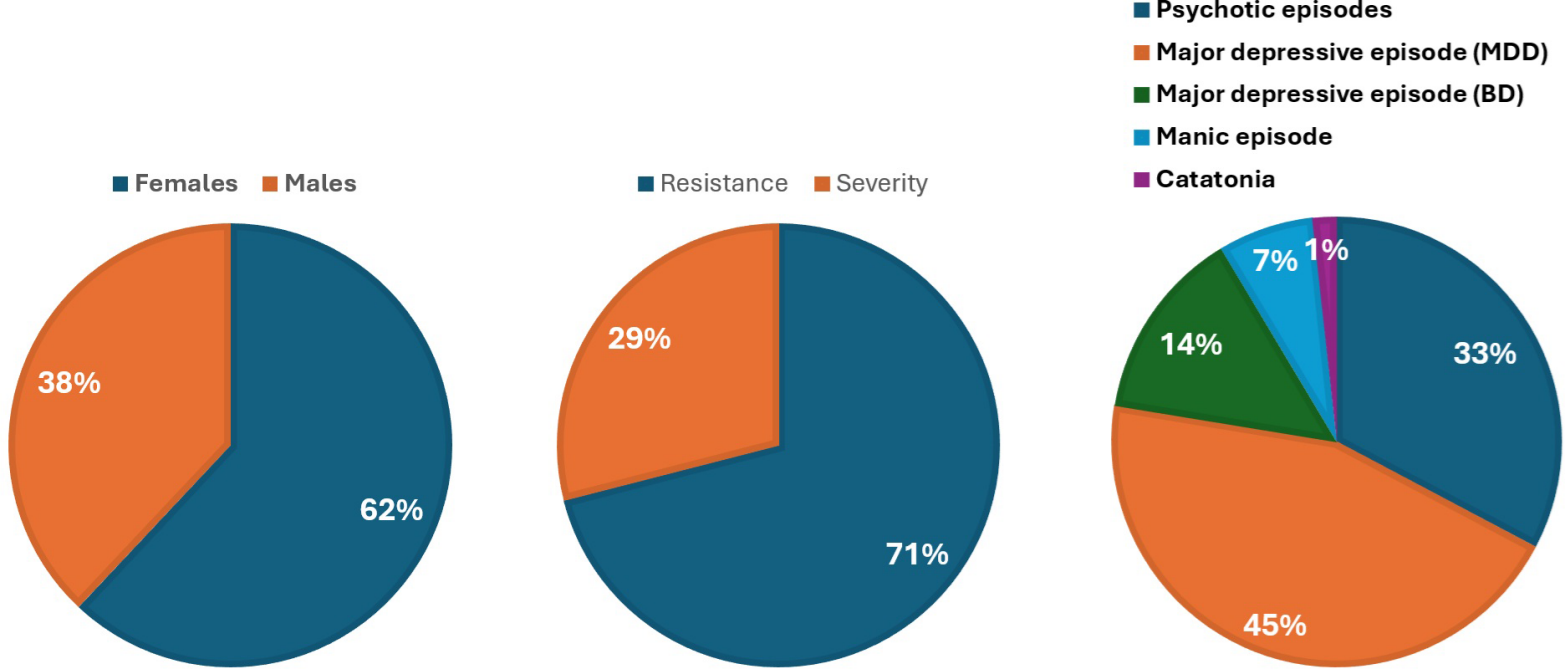
Illustration of some demographic and clinical characteristics of the study cohort. *BD: Bipolar Disorder; MDD: Major Depressive Disorder.* Percentages were rounded, with the smaller category subsequently corrected to achieve a total of 100 %.

The study included 58 patients (62.1% women) with a mean age of 47.8 ± 18.6 years. Mean disease duration was 17.8 ± 14.3 years from symptom onset and 14.5 ± 13.6 years from diagnosis. Diagnoses were 32.7% psychotic episodes (schizophrenia except one patient with a schizoaffective disorder), 44.8% unipolar depression, 13.8% bipolar depression, 6.9% manic episode, and 1.7% catatonia. ECT was indicated for treatment resistance in 70.7% and for severity in 29.2%, with severity due to catatonic features (10.3%), melancholic features (10.3%), or suicidal crisis (8.6%). These data are illustrated in **Figure 1**. The current episode had lasted 15.6 ± 12.2 weeks at assessment. Patients had experienced a mean of 3.2 ± 3.5 prior depressive episodes, 0.7 ± 2.4 manic episodes, 0.1 ± 1.0 hypomanic episodes, and 1.2 ± 2.6 psychotic episodes. The cumulative number of mood episodes averaged 4.4 ± 4.7 and that of psychiatric episodes 5.6 ± 4.5. Patients reported a mean of 0.3 ± 0.7 psychoactive substances consumptions and 0.4 ± 0.7 psychiatric comorbidities, with the combined addictive and psychiatric comorbidities averaging 0.6 ± 0.7. Psychiatric comorbidities were documented in 19% (obsessive-compulsive disorder 8.6%, eating disorder 8.6%, borderline personality disorder 3.3%, attention deficit and hyperactivity disorder 3.3%, autism spectrum disorder 1.7%). Substance consumption was reported in 24.1% (cannabis 5.2%, alcohol 12.1%, cigarettes 13.8%, benzodiazepine 1.7%). Among the initial 58 patients who started a RUL ECT, stimulation was switched to BT due to inadequate clinical response in 18 cases (31.0%).

The treatment profiles during the first ECT course (*n* = 58) were as follows: antidepressants (56.9%), antipsychotics (65.5%), clozapine (15.5%), lithium (25.9%), anticonvulsants (13.8%), pramipexole (13.8%), sedative antihistaminic treatments (1.7%) or benzodiazepines (6.9%). Antidepressants categories were as follows: 27.6% specific serotonin-norepinephrine reuptake inhibitors (n=13 venlafaxine and n=3 duloxetine), 22.4% presynaptic α2-adrenoceptor antagonist (mirtazapine), 12.1% specific serotonin reuptake inhibitors (n=4 sertraline, n=2 fluoxetine, n=1 escitalopram), 10.3% vortioxetine, 3.4% tricyclic antidepressants (clomipramine), 3.4% monoamine oxidase inhibitors (n=1 phenelzine, n=1 tranylcypromine), and 1.7% tetracyclic antidepressants (maprotiline).

The treatment profiles during the second ECT course (*n* = 18) were as follows: antidepressants (50.0%), antipsychotics (66.7%), clozapine (55.6%), lithium (33.3%), anticonvulsants (5.6%), pramipexole (27.8%), sedative antihistaminic treatments (0.0%) or benzodiazepines (0.0%). Antidepressants categories were as follows: 11.1% specific serotonin-norepinephrine reuptake inhibitors (n=1 venlafaxine and n=1 duloxetine), 16.7% presynaptic α2-adrenoceptor antagonist (mirtazapine), 5.6% specific serotonin reuptake inhibitors (fluoxetine), 11.1% vortioxetine, 5.6% tricyclic antidepressants (amitriptyline), and 16.7% monoamine oxidase inhibitors (n=1 phenelzine, n=1 selegiline, n=1 tranylcypromine).

During both ECT courses, no patient received a norepinephrine-dopamine reuptake inhibitor (i.e., bupropion).

The number of ECT sessions in the RUL and BT courses were 14.3 ± 7.1 and 14.7 ± 9.7, respectively. The mean charge at the first session was 172.6 ± 99.2 mC for RUL and 152.9 ± 82.3 mC for BT, increasing to 214.4 ± 125.8 mC and 236.2 ± 238.7 mC, respectively, with Δ charge from first to last session being 41.8 ± 76.5 mC and 83.3 ± 187.5 mC, respectively. Seizure durations were as follows: first session (RUL 64.1 ± 32.9 s; BT 63.2 ± 34.7 s) and last session (RUL 47.3 ± 22.1 s; BT 50.9 ± 36.7 s). Abrupt PIS was observed in 60.3% of RUL and 66.7% of BT sessions initially, increasing to 82.8% in RUL and remaining 66.7% in BT by the last session.

**Figure 2:**
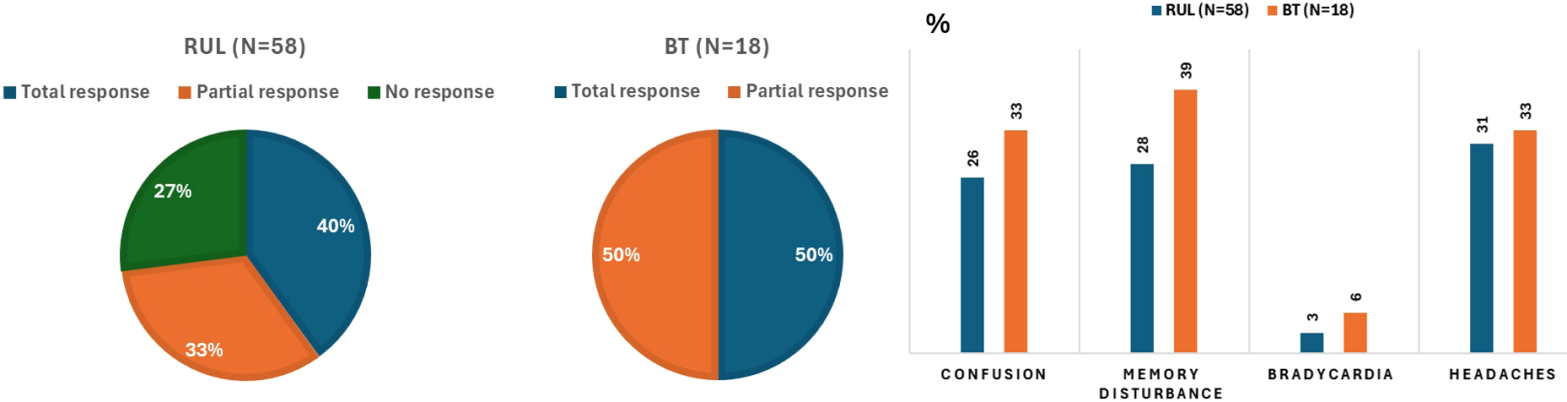
Efficacy and tolerability during the right unilateral (RUL) and bitemporal (BT) electroconvulsive therapy courses. Percentages were rounded, with the smaller category subsequently corrected to achieve a total of 100 %.

Total clinical response occurred in 39.7% of RUL patients and 50.0% of BT patients, with partial response in 32.8% (RUL) and 50.0% (BT), and no response in 27.6% (RUL) and 0.0% (BT). The cumulative clinical efficacy reached 55.2%. Good tolerability was reported in 50.0% of RUL and 44.4% of BT sessions. Memory disturbance, confusion, and headache were observed in 27.6%, 25.9%, and 31.0% of RUL sessions, and 38.9%, 33.3%, and 33.3% of BT sessions, respectively; bradycardia was rare (RUL 3.4%, BT 5.6%). Efficacy and tolerability data are illustrated in **Figure 2**.

### 3.2 Data comparison in the unswitched *vs*. switched ECT group

The primary analysis pooled diagnostic groups to increase the sample size and improve the statistical power. Given the diagnostic heterogeneity, patterns of response trajectories and tolerability following RUL ECT, and subsequent switching to BT ECT by diagnostic indication category are described in **Table 1** for exploratory purposes.

**Table 1:** Clinical response following right unilateral (RUL) electroconvulsive therapy (ECT) and switching to bilateral (BT) ECT by diagnostic indication category.

Diagnostic indication category	Clinical response following RUL, *n* (%)	Good tolerability following RUL, *n* (%)	Switch RUL→BT, *n* (%)
Psychotic episode (n=19)	5 (26.3%) Total response 6 (31.6%) Partial response 8 (42.1%) Non-response	9 (47.4%)	8 (42.1%)
Major depressive episode (Major depressive disorder) (n=26)	11 (42.3%) Total response 9 (34.6%) Partial response 6 (23.1%) Non-response	15 (57.7%)	8 (30.8%)
Major depressive episode (bipolar disorder) (n=8)	3 (37.5%) Total response 3 (37.5%) Partial response 2 (25.0%) Non-response	4 (50.0%)	2 (25.0%)
Manic episode (n=4)	3 (75.0%) Total response 1 (25.0%) Partial response	1 (25.0%)	0 (0.0%)
Catatonia (n=1)	1 (100.0%) total response	0 (0.0%)	0 (0.0%)

**Figure 3:**
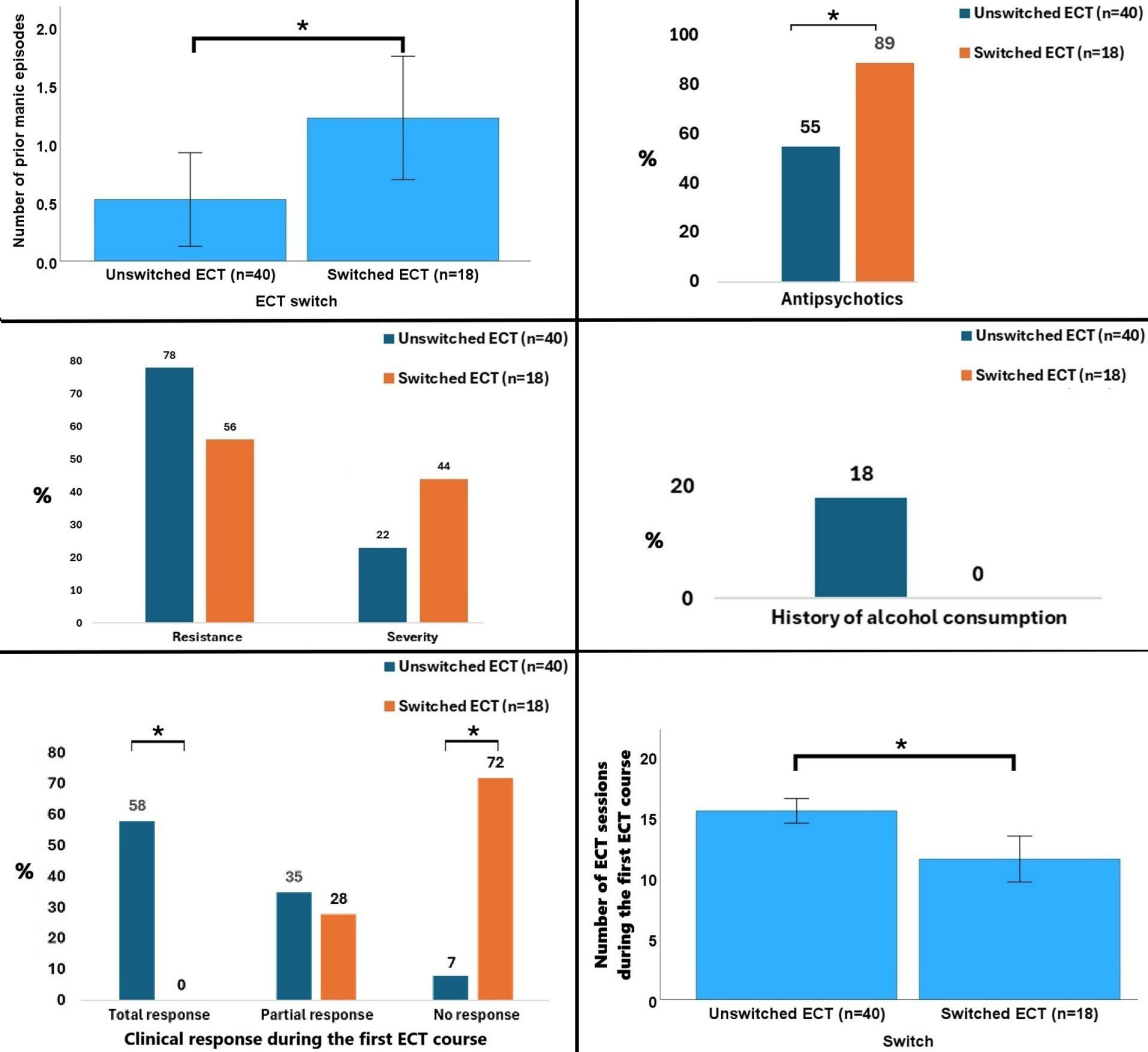
Data that significantly differed or tended to differ between patients who remained in the right unilateral treatment vs. those switched to bitemporal treatment. *p<0.05. Percentages were rounded, with the smaller category subsequently corrected to achieve a total of 100 %.

At baseline, patients who switched to the BT ECT (*n* = 18) had more previous manic episodes (1.2 ± 2.2 *vs*. 0.5 ± 2.5; p = 0.021, effect size *r* = 0.30) and higher antipsychotic use during RUL ECT (88.9% *vs*. 55.0%; p = 0.012) compared to those who pursued RUL ECT (*n* = 40). There was also a tendency toward higher frequency of ECT indication for severity (44.4% *vs*. 22.5%; p=0.089), lower frequency of ECT indication for resistance (55.6% *vs*. 77.5%; p = 0.089), and lower alcohol use (0.0% *vs*. 17.5%; p = 0.058) in the switched group. The only significant procedural difference was the number of sessions: patients who completed RUL ECT received on average 15.5 ± 6.4 sessions, whereas patients who switched to BT ECT completed 11.6 ± 8.0 sessions using the RUL setup before switching (p = 0.029, effect size *r* = 0.29). Regarding clinical outcomes, there were significant group differences in clinical efficacy during the first ECT course (p<0.001), with total response being significantly and exclusively observed in the unswitched group (57.5% *vs*. 0.0%, *post hoc* p<0.05) whereas non-response being significantly observed in the group who subsequently underwent the switch (7.5% *vs*. 72.2%, *post hoc* p<0.05) (**Figure 3**). This occurred in the lack of significant group differences in terms of sociodemographic variables, remaining clinical, stimulation/seizure and ECT-outcomes (tolerability during the RUL course, globally or individual side effects). All the data are summarized in **Table 2**.

**Table 2:** Baseline demographic and clinical characteristics, clinical outcomes and electroconvulsive therapy (ECT) variables of the first ECT course in patients continuing right unilateral (RUL) ECT versus those switched to bitemporal (BT) ECT. .

Variable	RUL (*n=*40)	RUL→BT (*n=*18)	*χ²/U*	P	Effect size
Age (years)	49.0 ± 20.8	45.1 ± 12.5	*U* = 339.00	0.724	0.05
Sex (females), *n* (%)	24 (60.0%)	12 (66.7%)	*χ²* = 0.23	0.628	–
Diagnostic categories, *n* (%)	11 (27.5%) Psychotic episode 18 (45.0%) Unipolar depression 6 (15.0%) Bipolar depression 4 (10.0%) Manic episode 1 (2.5%) Catatonia	8 (44.4%) Psychotic episode 8 (44.4%) Unipolar depression 2 (11.1%) Bipolar depression 0 (0.0%) Manic episode 0 (0.0%) Catatonia	*χ²* = 3.48	0.482	–
Indication for ECT, *n* (%)	31 (77.5%) Resistance 9 (22.5%) Severity	10 (55.6%) Resistance 8 (44.4%) Severity	*χ²* = 2.89	0.089	–
Disease duration since first symptoms (years)	17.7 ± 13.9	18.1 ± 15.3	*U* = 357.50	0.966	0.01
Duration of illness since diagnosis (years)	14.5 ± 13.4	14.6 ± 14.5	*U* = 351.50	0.886	0.02
Duration of current episode (weeks)	14.4 ± 12.1	18.3 ± 12.4	*U* = 426.00	0.266	0.15
Previous depressive episodes	3.5 ± 3.6	2.7 ± 3.4	*U* = 301.00	0.311	0.13
Previous manic episodes	0.5 ± 2.5	1.2 ± 2.2	*U* = 438.00	**0.021**	0.30
Previous hypomanic episodes	0.5 ± 1.0	0.2 ± 0.7	*U* = 326.50	0.392	0.11
Previous psychotic episodes	1.2 ± 2.6	1.1 ± 2.7	*U* = 359.00	0.983	0.00
Cumulative mood/psychotic episodes	5.7 ± 4.5	5.2 ± 4.6	*U* = 349.00	0.692	0.05
Number of psychiatric and addictive comorbidities	0.6 ± 0.8	0.6 ± 0.6	*U* = 364.00	0.940	0.01
Any psychiatric comorbidities, *n* (%)	6 (15.0%)	5 (27.8%)	*χ²* = 1.32	0.251	–
Eating disorders, *n* (%)	3 (7.5%)	2 (11.1%)	*χ²* = 0.21	0.650	–
Obsessive-compulsive disorder, *n* (%)	4 (10.0%)	1 (5.6%)	*χ²* = 0.31	0.577	–
Any substance consumption, *n* (%)	11 (27.5%)	3 (16.7%)	*χ²* = 0.80	0.372	–
Cannabis consumption, *n* (%)	2 (5.0%)	1 (5.6%)	*χ²* = 0.01	0.930	–
Alcohol consumption, *n* (%)	7 (17.5%)	0 (0%)	*χ²* = 3.58	0.058	–
Smoking consumption, *n* (%)	6 (15%)	2 (11.1%)	*χ²* = 0.16	0.691	–
Antipsychotics, *n* (%)	22 (55.0%)	16 (88.9%)	*χ²* = 6.31	**0.012**	**-**
Clozapine, *n* (%)	7 (17.5%)	2 (11.1%)	*χ²* = 0.39	0.534	–
Antidepressants, *n* (%)	23 (57.5%)	10 (55.6%)	*χ²* = 0.02	0.890	–
Lithium, *n* (%)	11 (27.5%)	4 (22.2%)	*χ²* = 0.18	0.671	–
Anticonvulsants, *n* (%)	6 (15.0%)	2 (11.1%)	*χ²* = 0.16	0.691	–
Pramipexole, *n* (%)	6 (15.0%)	2 (11.1%)	*χ²* = 0.16	0.691	–
Sedative antihistaminic treatments, *n* (%)	1 (2.5%)	0 (0.0%)	*χ²* = 0.46	0.499	–
Benzodiazepines, *n* (%)	3 (7.5%)	1 (5.6%)	*χ²* = 0.07	0.787	–
Total number of sessions (number)	15.5 ± 6.4	11.6 ± 8.0	*U* = 230.00	**0.029**	0.29
Δ charge first-last sessions (mC)	40.5 ± 60.2	44.6 ± 106.4	*U* = 300.50	0.302	0.14
Charge first session (mC)	181.0 ± 111.4	154.1 ± 63.3	*U* = 310.50	0.382	0.11
Charge last session (mC)	221.5 ± 121.3	198.7 ± 137.7	*U* = 286.00	0.206	0.17
Seizure duration first session (s)	61.3 ± 31.8	70.2 ± 35.4	*U* = 427.00	0.260	0.15
Seizure duration last session (s)	46.7 ± 20.1	48.7 ± 26.4	*U* = 374.50	0.807	0.03
Postictal suppression first session, *n* (%)	22 (55.0%)	13 (72.2%)	*χ²* = 1.54	0.215	–
Postictal suppression last session, *n* (%)	35 (87.5%)	13 (72.2%)	*χ²* = 2.03	0.154	–
Clinical efficacy, *n* (%)	23 (57.5%) Total response* 14 (35.0%) Partial response 3 (7.5%) Non-response*	0 (0%) Total response* 5 (27.8%) Partial response 13 (72.2%) Non-response*	*χ²* = 29.40	**<0.001**	**-**
Good tolerability, *n* (%)	21 (52.5%)	8 (44.4%)	*χ²* = 0.32	0.570	–
Memory disturbance, *n* (%)	11 (27.5%)	5 (27.8%)	*χ²* = 0.00	0.983	–
Post-ictal confusion, *n* (%)	12 (30.0%)	3 (16.7%)	*χ²* = 1.15	0.283	–
Headache, *n* (%)	13 (32.5%)	5 (27.8%)	*χ²* = 0.13	0.719	–
Bradycardia during the session, *n* (%)	1 (2.5%)	1 (5.6%)	*χ²* = 0.35	0.555	–

Significant p values are bolded. *p< 0.05 in post-hoc tests.

Binary logistic regression was conducted to examine the association between some baseline clinical and sociodemographic variables (independent variables) and the likelihood of switching from RUL to BT ECT (dependent variable: switch yes/no). Only baseline variables that differed significantly in group comparisons were entered into the model, namely the number of previous manic episodes and the frequency of antipsychotic use. The best regression model retained only the frequency of antipsychotic use and was statistically significant, χ² (1, N = 58) = 7.12, p = 0.008. This model explained 16.3% of the variance in switching (Nagelkerke R²) and correctly classified 69.0% of cases. Patients receiving antipsychotic medication were significantly more likely to be switched from RUL to BT ECT than those not receiving antipsychotics (OR = 6.55, 95% CI [1.58, 44.94]). The number of previous manic episodes was not a significant predictor of the switching strategy.

### 3.3 Data comparison in the switched group during the right unilateral *vs*. bitemporal course

**Figure 4:**
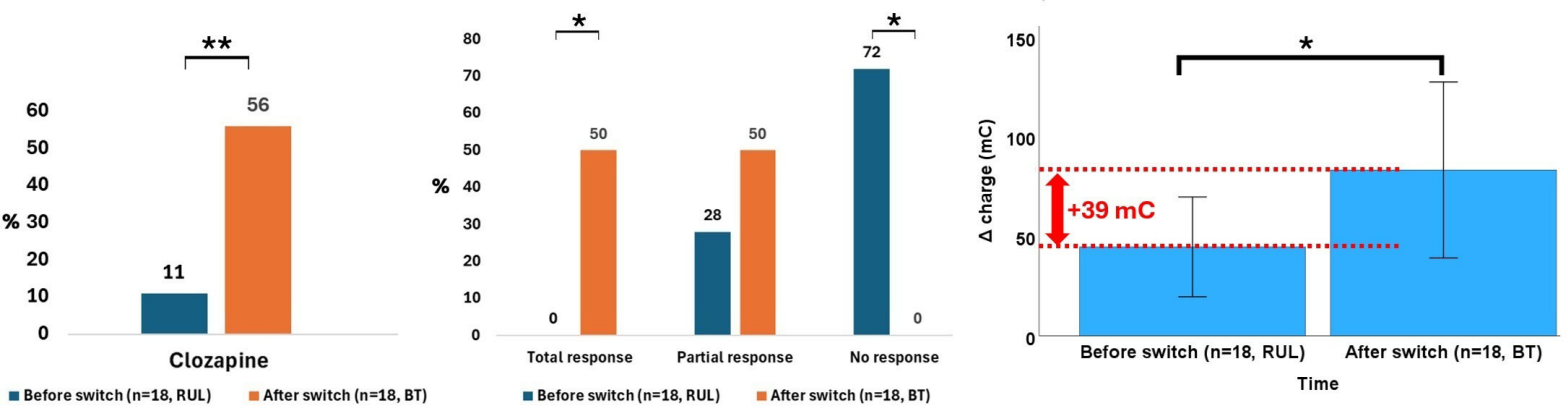
Data that significantly differed in patients who underwent a switch from right unilateral (RUL) to bitemporal (BT) electroconvulsive therapy (ECT).*p<0.05, **p<0.01.

In the subgroup of patients who underwent both RUL and BT stimulation (n = 18), the Δ charge was significantly greater during BT (+ 38.7 mC, p = 0.049, effect size *r* = 0.46), in the absence of significant differences in the number of ECT sessions, seizure duration at the first or last session, PIS abruptness at either session, or charge at the first or last session. Regarding treatments, clozapine use increased by + 44.5% from the RUL to the BT course (p = 0.005), while other medication did not. A significant difference in efficacy emerged (p < 0.001): Total response occurred only during BT course (50.0% *vs*. 0.0%, *post hoc* p < 0.05), while non-response occurred only during RUL course (72.2% *vs*. 0.0%, *post hoc* p < 0.05). Tolerability was comparable (p > 0.999), with nonsignificant increases in confusion (+16.7%, p = 0.248), memory disturbance (+11.3%, p = 0.480), and headaches (+5.5%, p = 0.717), and no change in bradycardia (p > 0.999). The main findings are illustrated in **Figure 4**. The whole results of this analysis are summarized in **Table 3**.

**Table 3:** Clinical characteristics, clinical outcomes and electroconvulsive therapy (ECT) variables of the patients who underwent a switch from right unilateral (RUL) ECT to bitemporal (BT) ECT.

Variables	RUL ECT course (*n=*18)	BT ECT course (*n=*18)	*χ²/W*	P	Effect size
Antipsychotics, *n* (%)	16 (88.9%)	12 (66.7%)	*χ²* = 2.57	0.109	–
Clozapine, *n* (%)	2 (11.1%)	10 (55.6%)	*χ²* = 8.00	**0.005**	**-**
Antidepressants, *n* (%)	10 (55.6%)	9 (50.0%)	*χ²* = 0.11	0.738	–
Lithium, *n* (%)	4 (22.2%)	6 (33.3%)	*χ²* = 0.55	0.457	–
Anticonvulsants, *n* (%)	2 (11.1%)	1 (5.6%)	*χ²* = 0.36	0.546	–
Pramipexole, *n* (%)	2 (11.1%)	5 (27.8%)	*χ²* = 1.60	0.206	–
Antihistaminic, *n* (%)	0 (0.0%)	0 (0.0%)	–	–	–
Benzodiazepines, *n* (%)	1 (5.6%)	0 (0.0%)	*χ²* = 1.03	0.310	–
Total number of sessions (number)	11.6 ± 8.0	14.7 ± 9.7	*W* = 93.50	0.421	0.19
Δ charge first-last sessions (mC)	44.6 ± 106.4	83.3 ± 187.5	*W* = 106.00	**0.049**	0.46
Charge first session (mC)	154.1 ± 63.3	152.9 ± 82.3	*W* = 64.00	0.553	0.14
Charge last session (mC)	198.7 ± 137.7	236.2 ± 238.7	*W* = 93.50	0.421	0.19
Seizure duration first session (s)	70.2 ± 35.4	63.2 ± 34.7	*W* = 74.00	0.616	0.12
Seizure duration last session (s)	48.7 ± 26.4	50.9 ± 36.7	*W* = 73.50	0.887	0.04
Postictal suppression first session, *n* (%)	13 (72.2%)	12 (66.7%)	*χ²* = 0.13	0.717	–
Postictal suppression last session, *n* (%)	13 (72.2%)	12 (66.7%)	*χ²* = 0.13	0.717	–
Clinical efficacy, *n* (%)	0 (0.0%) Total response* 5 (27.8%) Partial response 13 (72.2%) Non-response*	9 (50.0%) Total response* 9 (50.0%) Partial response 0 (0.0%) Non-response*	*χ²* = 23.14	**<0.001**	**-**
Good tolerability, *n* (%)	8 (44.4%)	8 (44.4%)	*χ²* = 0.00	>0.999	–
Memory disturbance, *n* (%)	5 (27.8%)	7 (38.9%)	*χ²* = 0.50	0.480	–
Post-ictal confusion, *n* (%)	3 (16.7%)	6 (33.3%)	*χ²* = 1.33	0.248	–
Headache, *n* (%)	5 (27.8%)	6 (33.3%)	*χ²* = 0.13	0.717	–
Bradycardia during the session, *n* (%)	1 (5.6%)	1 (5.6%)	*χ²* = 0.00	>0.999	–

Significant p values are bolded. *p< 0.05 in post-hoc tests.

### 3.4 Supplementary analysis of efficacy and tolerability in each time course

Following the RUL ECT course, the number of prior manic episodes differed significantly across efficacy groups (p = 0.022, effect size *E^2R^* = 0.13). *Post hoc* analysis showed that non-responders had a higher number of prior manic episodes compared to partial responders (1.4 ± 2.3 *vs*. 0.0 ± 0.0, p = 0.020), while no differences emerged between total responders and either group. Patients with good tolerability during RUL ECT were older (53.6 ± 17.5 *vs*. 42.0 ± 18.1, p = 0.013, effect size *r* = 0.32), had longer disease duration since first symptoms (21.8 ± 14.9 *vs*. 13.8 ± 12.6, p = 0.031, effect size *r* = 0.28) and since diagnosis (17.5 ± 14.6 *vs*. 11.5 ± 12.1, p = 0.042, effect size *r* = 0.27), and had experienced more prior depressive episodes (4.4 ± 4.2 *vs*. 2.1 ± 2.2, p = 0.037, effect size *r* = 0.27) and psychiatric episodes overall (6.7 ± 4.6 *vs*. 4.4 ± 4.2, p = 0.028, effect size *r* = 0.29). Following the BT ECT course, no significant differences were observed among efficacy groups, although clozapine intake tended to be lower in total responders than partial responders (33.3% *vs*. 77.8%, p = 0.058). Better tolerability during BT ECT was associated with the absence of psychiatric comorbidities, both in frequency (0.0% *vs*. 50.0%, p = 0.019) and number (0.0 ± 0.0 *vs*. 0.8 ± 0.9, p = 0.083, effect size *r* = 0.53).

## 4 Discussion

### 4.1 Summary of findings

This retrospective study examined clinical outcomes and switching strategies in titrated ECT with an initial RUL course followed by BT course in cases of insufficient response in a French cohort of adults with psychiatric disorders. About one-third of patients required a switch, with overall efficacy rates around 40% after RUL ECT and exceeding 50% when both courses were considered, while tolerability remained comparable despite a greater charge increase during BT ECT. Compared to patients who completed RUL ECT alone, those requiring BT ECT were more likely to have a history of manic episodes and to receive antipsychotic treatments, with trends toward greater severity, lower treatment resistance, and less alcohol use. Among switched patients, BT ECT was associated with a higher Δ charge and more frequent clozapine prescription. Additional analyses suggested that older age, longer illness duration, and more prior depressive episodes were associated with better tolerability in RUL, while fewer psychiatric comorbidities were associated with better tolerability in BT ECT. Regarding efficacy, fewer prior manic episodes differentiated partial from non-responders in RUL ECT, and lower clozapine prescription was found in total compared to partial responders in BT ECT.

### 4.2 Between-group comparison: patients completing RUL *vs*. patients switching to BT ECT

The switched group had a higher number of previous manic episodes, greater antipsychotic use, and a tendency toward ECT initiation for symptom severity rather than treatment resistance. Antipsychotic use was a significant predictor of switching from RUL to BT ECT. These findings highlight potential clinical factors associated with poorer RUL efficacy. While high-dose RUL ECT can sometimes approach the efficacy of BT ECT (2, 5, 8), our findings support the notion that patients with more severe or complex illness trajectories may benefit less from cognitive-sparing strategies and may ultimately require BT placement. Reviews and meta-analyses suggest that although high-dose RUL can approximate BT ECT efficacy, its effectiveness is limited in certain subgroups of patients (50; CANMAT, 1, 13). BT stimulation may achieve faster or more robust responses, whereas some patients undergoing RUL ECT experience slower or lower efficacy (1, 5–15). Consistently, in our cohort, non-responders to RUL were successfully rescued with BT stimulation. Earlier studies have linked treatment resistance to poorer ECT outcomes (51–53), and recent trials show that RUL-to-BT switching occurs more often in highly resistant patients who also seem to require more sessions (32). These findings support earlier ECT intervention in less resistant patients to reduce treatment burden and limit the need for BT switching, thereby minimizing cognitive side effects. Conversely, in one of their sequential ECT studies, Sackeim and colleagues suggest that efficacy improvements in initial non-responders may stem from prolonging treatment rather than electrode switching to a bilateral setup, since their patients improved regardless of the first ECT course (unilateral or bilateral) (4). Further, their second BT course was associated with more pronounced cognitive symptoms, highlighting the need for randomized trials to clarify the relative contributions of extended treatment versus electrode placement (4).

From a mechanistic perspective, differences in RUL and BT ECT efficacy may reflect distinct neurobiological mechanisms. Bilateral ECT could induce a stronger activation of diencephalic, hypothalamic, and prefrontal networks, which are implicated in regulating vegetative and mood symptoms (54–56), and may produce higher charge density in these regions as well as more symmetric cortical seizure patterns compared to RUL (57–59). High dose RUL can recruit broader cortical regions compared to a low dose regimen, thereby approximating the effects of bilateral electrode placement (60–64).

Interestingly, and somewhat counterintuitively, patients who required a switch in our study tended to have a lower prevalence of alcohol use compared to those who continued RUL ECT. Evidence linking substance use to ECT outcomes is limited and mixed. Some studies suggest a history of alcohol use may be associated with a better clinical response in patients with MDD or schizophrenia, potentially via modulation of the glutamatergic-GABAergic (excitatory–inhibitory) balance (65, 66), whereas others report poorer or unchanged outcomes in patients with mood disorders and comorbid alcohol or substance use disorders (67–69). In our inpatient setting, this finding could be an artifact of abstinence from alcohol, which may have partly contributed to clinical improvement and potentially reduced the need to switch to BT ECT. This was described in previous work with adolescents and young adults with depression (70).

### 4.3 Within-group comparison: RUL *vs*. BT ECT courses in patients who underwent switch

The within-patient comparison in the switched subgroup highlights the clinical value of BT ECT in initially refractory cases, with remission occurring exclusively during the BT course in half of these patients, whereas non-response was confined to the RUL course. This underscores the utility of sequential strategies to maximize efficacy while limiting exposure to the higher cognitive risks of BT. Although seizure quality and session numbers did not differ significantly, BT ECT was associated with greater Δ charge and increased clozapine use, reflecting the pharmacological complexity and severity of cases requiring optimization of neuromodulation and pharmacological protocols to achieve a clinical response. Clozapine, a marker of treatment refractoriness, illustrates the multimodal approach of combining neurostimulation and pharmacotherapy, consistent with guideline recommendations (71). Its higher use in patients undergoing BT ECT may reflect an augmentation strategy aimed at optimizing treatment outcomes.

### 4.4 Supplementary analysis: comparing outcomes according to efficacy and tolerability

Supplementary analyses identified clinical factors associated with ECT outcomes. However, these findings are exploratory and should be interpreted with caution. Efficacy was associated with a lower number of prior manic episodes in the RUL course, and with lower clozapine prescription in the BT course. Fewer prior manic episodes may indicate predominantly depressive illness, reflecting less severe or even less resistant disease. Similarly, clozapine prescription may reflect disease severity and treatment resistance, and the observed association should be interpreted with caution; it likely reflects a bias due to confounding by indication rather than a causal effect of the medication on ECT outcomes. As such, patients with more severe or resistant illnesses are more likely to receive clozapine. Prior studies have identified predictors of good ECT response in depression, including short episode duration (31, 72–78), absence of chronic depression or dysthymia (73), preserved insight (74), low pharmacological resistance (32, 73, 75, 77, 79), low disability burden (80), and absence of psychiatric comorbidities (79, 80). Evidence regarding bipolar depression is mixed, with efficacy reported as higher (30, 31, 79, 81), lower (74, 82), or comparable (30, 83, 84) to unipolar depression. Some studies suggest bipolar depression responds more rapidly (83, 85–87) and may require fewer sessions (30, 85, 88). Baseline symptom severity shows inconsistent associations with response (29, 77, 80, 82, 84, 89). Additional clinical correlates include psychomotor symptoms (76, 78, 90, 91), which may underlie the association between older age and efficacy in unipolar depression (76, 90), and psychotic features, generally associated with better outcomes (77, 78, 90; for reviews see 28; meta-analysis by 29) though not consistently (74, 75). Evidence regarding melancholic features is mixed (28, 29, 75). High severity of suicidality has also been highlighted as a predictor of good response (for reviews see 28). Regarding age, several studies and reviews have reported a positive association between age and ECT efficacy (28, 31, 69, 73, 78, 79, 84, 92, 93; meta-analysis by 29), whereas others found no effect (75, 76). Beyond efficacy, controversial findings concern cognitive outcomes: older adults were reported to be less likely to experience cognitive decline and more likely to exhibit cognitive improvement following ECT compared with younger patients (94). However, older age was also associated with higher rates of transient cardiac arrhythmia during treatment (93, 95), and in other studies, it was associated with more cognitive side effects (95, 96).

Tolerability during the RUL course was associated with older age, longer illness duration and a higher recurrence rate of psychiatric episodes. This latter finding may reflect the familiarity with and acceptance of psychiatric interventions, consistent with evidence on the influence of patients’ expectations on ECT outcomes (97). Repeated exposure to interventions throughout a chronic illness could mitigate anxiety and uncertainty, enabling patients to approach ECT with greater composure. Moreover, individuals with more severe or recurrent illness may perceive ECT as necessary and effective, which may further enhance tolerability. During the BT course, tolerability was higher in patients with fewer psychiatric comorbidities, suggesting that clinical complexity contributes not only to treatment resistance but also to vulnerability to side effects. Supporting this, patients with comorbid personality disorders or traits have been reported to show higher rates of side effects and relapse following ECT compared with those without such comorbidities (98). Additionally, polypharmacy - common among patients with multiple comorbidities - may further increase the risk of adverse reactions.

### 4.5 Study strengths, limitations, and future perspectives

Several limitations of this study should be acknowledged. First, the small sample size - particularly in the switched group - may have reduced statistical power and may limit the generalizability of the findings. For the same reason, despite the numerous outcomes assessed, no statistical correction for multiple testing was applied, which may increase the risk of type I error (i.e., false positives). Similarly, stratified analyses were not conducted. Second, the absence of long-term follow-up prevents conclusions regarding relapse risk and remission durability. Third, outcomes relied on chart reviews without systematic neuropsychological, neuroimaging, or neurophysiological assessments. The absence of validated symptom severity scales to characterize the study cohort (e.g., Montgomery-Åsberg Depression Rating Scale or Hamilton Depression Rating Scale for depression, Positive and Negative Symptoms Scale for schizophrenia) limits the comparability of the present findings with previous studies. Additionally, clinical efficacy was categorized based on treating clinicians’ judgment which may introduce inter-rater variability and may carry potential misclassification risk inherent to chart-based categorical outcomes. Assessments were also performed by the treating clinicians rather than independently, reflecting real-world practice but potentially introducing another source of bias. In addition, symptom dimensions were not analyzed, although some work suggests specific symptom domains predict ECT response in depression (99, 100). Moreover, these limitations also apply to tolerability, which was dichotomized based on subjective complaints or clinician assessment rather than graded by severity using standardized tools (e.g., neuropsychological testing for cognitive side effects such as the ElectroConvulsive therapy Cognitive Assessment tool; 101). This approach may have obscured clinically meaningful differences in the severity or persistence of side effects. Furthermore, concerning the seizure quality induced by ECT, despite regular training and supervision to ensure EEG readings are standardized, clinician bias cannot be entirely excluded. Future research would benefit from incorporating standardized, validated rating instruments and independent evaluations to improve reliability and comparability.

The current findings underscore the need for multimodal approaches integrating clinical, biological, neurophysiological and neuroimaging predictors to improve patient stratification and guide personalized ECT strategies in large, prospective, controlled cohorts with a long-term follow-up. Increasing interest in biological predictors includes microRNA signatures linked to inflammatory pathways (102), baseline inflammatory markers (103), and kynurenine pathway metabolites (104). Other promising markers involve brain functional connectivity patterns or metabolites, plasma homovanillic acid and Tumor necrosis factor-α, and specific genetic polymorphisms (28, 105), although current methods remain below translational thresholds.

## Data Availability

The raw data supporting the conclusions of this article will be made available by the authors, without undue reservation.
